# Internet-assisted cognitive behavioral therapy with telephone coaching versus an educational control for antenatal depression: protocol for a randomized controlled trial with population-based screening

**DOI:** 10.3389/fpsyt.2025.1604352

**Published:** 2025-11-03

**Authors:** Andre Sourander, Tarja Korpilahti-Leino, Terja Ristkari, Tarja Koffert, Bianca Arrhenius, Tiia Ståhlberg, Susanna Hinkka-Yli-Salomäki, Mari Berglund, Subina Upadhyaya, Wan Mohd Azam Wan Mohd Yunus, Atte Sinokki, Riku Hägg, Altti Marjamäki, Iida Kankaanranta, Johanna Palmroth, Saana Sourander, Anna Zadkova, Linda Casagrande, Yuko Yamada, Satu Karjalainen, Hanna-Maria Matinolli, Miika Vuori

**Affiliations:** ^1^ Department of Child Psychiatry, University of Turku and Turku University Hospital, Turku, Finland; ^2^ Research Centre for Child Psychiatry, University of Turku, Turku, Finland; ^3^ INVEST Research Flagship Center, University of Turku, Turku, Finland; ^4^ Department of Adolescent Psychiatry, Turku University Hospital, Turku, Finland; ^5^ Faculty of Social Sciences and Humanities, Universiti Teknologi Malaysia, Johor, Malaysia; ^6^ Research Unit, The Social Insurance Institution of Finland (Kela), Helsinki, Finland

**Keywords:** antenatal depression, cognitive behavioral therapy, digital intervention, perinatal depression, telephone coaching

## Abstract

**Background:**

Up to 15% of pregnant women suffer from antenatal depression, and there is mounting evidence that the consequences can be devastating for both the woman and her child. Identifying effective treatments is crucial to avoiding the harmful consequences of unrecognized and untreated depression.

**Methods:**

This ongoing research project evaluates the efficacy of the *Stronger Together* internet-assisted cognitive behavioral therapy (iCBT) with telephone coaching for antenatal depression. It is a large, population-based, two-parallel group, randomized controlled trial. The overarching hypothesis is that iCBT programs are easy to access and affordable and will reduce self-reported symptoms of antenatal depression better than an educational control. The intervention group is also expected to report fewer anxiety symptoms after treatment than the control group. The protocol follows the Standard Protocol Items: Recommendations for Interventional Trials (SPIRIT) guidelines. The study population consists of all Finnish- or Swedish-speaking women, aged ≥18 years, who attend maternity health check-ups at 13–18 weeks of gestation in selected areas of Finland. The aim is to recruit all women who exceed 10 points on the Edinburgh Postnatal Depression Scale and meet other eligibility criteria. Other measures used are the Beck Depression Inventory-II, the Generalized Anxiety Disorder 7-Item Scale, the Pregnancy-Related Anxiety Questionnaire-Revised, the Social Phobia Inventory, and the Perceived Stress Scale. In addition, biological samples (maternal sera and buccal cells) are collected to explore possible moderators for the treatment response. The primary data are collected at baseline and 11 weeks after randomization. The women are randomized 1:1 to the *Stronger Together* iCBT intervention, which combines seven-weekly themes on a digital platform with weekly telephone coaching, or the educational control group. The CBT components include psychoeducation, behavioral activation, coping with social relationships, cognitive restructuring, and preventing setbacks. The educational control group receives psychoeducational material about wellbeing during pregnancy. Both groups continue to receive standard treatment and maternity health check-ups.

**Discussion:**

We hypothesize that the *Stronger Together* iCBT intervention will reduce self-rated symptoms of antenatal depression, general and pregnancy-related anxiety, social phobia, and stress. The intervention may offer an accessible and effective treatment for depressed pregnant women.

**Clinical Trial Registration:**

ClinicalTrials.gov, identifier ID NCT04223115, Date of first registration: January 10, 2020.

## 1 Introduction

Antenatal depression is a significant public health problem: as many as 10%–15% of pregnant women report depressive symptoms (1–3). Antenatal depression has been associated with preterm birth and low birth weight (4, 5), postnatal depression (6), and attachment difficulties between mother and infant (7). Previous studies on the long-term impact of maternal perinatal depression have also reported increased risks for behavioral problems, learning difficulties, and depression in offspring (8). It is crucial to provide empirically supported interventions at early time points, as most depressive symptoms emerge during pregnancy rather than after delivery (9). Early psychosocial interventions are also important because of concerns that selective serotonin reuptake inhibitors during pregnancy may be harmful to offspring development (10, 11).

Evidence-based psychosocial treatments, such as cognitive behavioral therapy (CBT) and interpersonal psychotherapy, are the first-line treatment options for antenatal depression (12, 13). A meta-analysis of 6,270 participants from 43 trials examined the efficacy of psychosocial treatments for depression during pregnancy. The overall effect size was moderate (g = 0.67, 95% confidence interval 0.45–0.89) (14). This finding was in line with the efficacy of psychosocial treatments for adult depression in general (15). Studies have not observed differences between the main types of psychosocial treatments and how they are delivered for depression (15, 16). Consequently, psychotherapies are also feasible for pregnant women, regardless of whether they are delivered face-to-face, individually, or in groups (13, 17). Digitally delivered internet-assisted CBT (iCBT) reduces logistical and practical barriers, including childcare, transport, and scheduling difficulties; meta-analyses have found significant treatment effects for general and perinatal depression (14, 15, 18). However, most previous studies have focused on the postpartum period, and the evidence for antenatal interventions is limited (19–22).

Previous randomized controlled trials (RCTs) on iCBT for antenatal depression have reported promising efficacy for reducing core symptoms of depression (21, 23, 24) with high acceptability of the iCBT interventions (21, 23). Attrition rates have been lower in guided than unguided iCBTs (18, 19, 23), and guided programs had higher effect sizes (19). However, studies on iCBT for antenatal depression have relied on small sample sizes and measured outcomes with self-reports only, posing a risk for overall assessment bias (19). No previous evidence exists of the effectiveness of iCBT for symptoms of antenatal depression in pregnant women screened at the population level. Larger population-based studies are needed to replicate previous findings from smaller studies and to identify effective treatment options for pregnant women whose mental health problems impact not only themselves but their offspring as well.

The aim of this study is to examine the efficacy of the *Stronger Together* iCBT intervention for symptoms of antenatal depression in adult pregnant women in Finland. An ideal antenatal iCBT intervention should facilitate and integrate service systems so that symptoms of antenatal depression are identified early and women have easy access to empirically supported treatments. The objectives of this study are to evaluate symptoms of antenatal depression among adult pregnant women in Finland using population-based screening, evaluate the efficacy of the Stronger Together iCBT intervention for treating symptoms of antenatal depression, and examine possible treatment moderators. The primary hypothesis is that the iCBT intervention will reduce self-reported symptoms of antenatal depression, measured by the Edinburgh Postnatal Depression Scale (EPDS), compared to the educational information (allocation ratio 1:1). The secondary hypothesis is that the iCBT intervention group will report fewer symptoms of generalized anxiety, pregnancy-related anxiety, and personal stress after treatment than the educational control group. This hypothesis is based on the literature; psychosocial interventions for perinatal depression have been found to be effective also for anxiety and stress (12).

## 2 Methods

### 2.1 Study type and design

**Figure 1:**
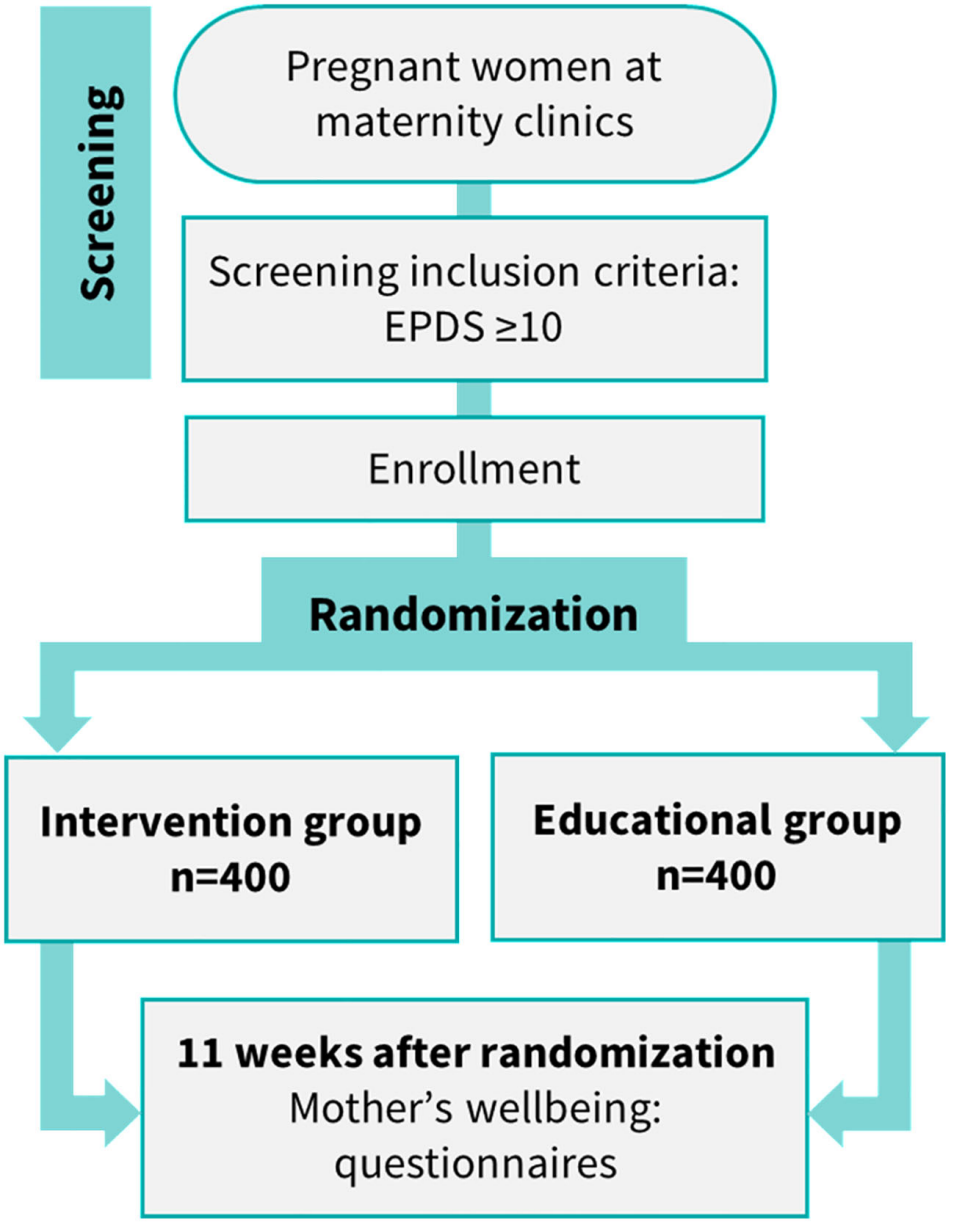
Flowchart of the RCT study. RCT, randomized controlled trial.

This study is a two-parallel group RCT. The study subjects and intervention coaches are open to the treatment code, but the statistician and researchers stay blinded until a blind review is conducted for the primary and secondary outcome variables. Blinding is an established approach in clinical trials to minimize the risk of performance and detection bias. Clinical trial guidelines recommend that statisticians remain blinded to allocation prior to the final analysis (25). In this RCT, pregnant women with depressive symptoms are randomized 1:1, by study site, into the *Stronger Together* iCBT intervention with telephone coaching or the educational control group. Both groups also receive standard treatment, according to established regional standard care guidelines, and this may involve therapy and medication. However, ongoing psychotherapy at recruitment is an exclusion criterion (**Table 1**). The study outline is presented in **Figure 1**.

**Table 1:** Inclusion and exclusion criteria.

Inclusion	Exclusion
- Age ≥ 18 years - Fluent in written and spoken Finnish or Swedish - Access to computer or mobile phone with internet/internet literacy - 12–22 weeks pregnant - EPDS score of ≥10 at screening and ≥9 at baseline	- Lifetime history of psychotic disorders, including schizophrenia, schizoaffective disorder, bipolar disorder, and psychotic depression - Active suicidal ideation - Severe substance abuse or dependence - Active ongoing psychotherapy - Already participating in another intervention study to treat the symptoms of antenatal depression - Expecting multiple births

EPDS, Edinburgh Postnatal Depression Scale.

### 2.2 Recruitment

Finnish maternity clinics provide regular check-ups 11–15 times during pregnancy to monitor the wellbeing of the fetus, the pregnant woman, and the whole family. Up to 99.8% of pregnant women in Finland use these services each year (26). This RCT consists of all the women attending regular maternity check-ups in the Wellbeing Services Counties of Southwest Finland, Central Ostrobothnia, and Siun Sote, and the cities of Espoo, Tampere, Pori, and Oulu. These areas account for approximately 14,000 live births per year, which is nearly one-third of the infants born in Finland each year (27). The women are screened using the EPDS, which is one of the most widely studied and validated self-report instruments for depressive symptoms during the perinatal period (28). Finnish national care guidelines state that the EPDS should be routinely used during health check-ups at 13–18 weeks of gestation.

All the women attending maternity clinics in the study areas are given an envelope during the first face-to-face health visit at 8–10 weeks of gestation. This includes an information letter, an informed consent document, and the EPDS questionnaire. Healthcare professionals also give the women verbal information about the ongoing study. The women are asked to complete the forms by the next clinic check-up at 13–18 weeks and provide informed consent if the research team is allowed to contact them about taking part in the RCT. The women are also provided with contact details for the study team in case they have any questions.

The informed consent forms and the EPDS questionnaires are collected during the routine check-up at 13–18 weeks. The women can fill in the forms during their visit if they have forgotten them. Scores of 10 points or more on the EPDS scale of 0 to 30 indicate possible depression. Those who have reached these scores and agreed to be contacted by the study group are invited to undergo an eligibility assessment for the RCT. As the EPDS form is routinely used by maternity health clinics, any symptoms are also discussed with the public health nurses working in the clinics. The public health nurses can contact the research group if they have any questions. The study coaches contact the public health nurses, regularly supporting them with the recruitment and other possible matters. Recruitment in the study is still ongoing in all participating areas.

### 2.3 Eligibility criteria

The study coaches call the women to introduce the study in more detail and screen them for eligibility, according to the inclusion and exclusion criteria in **Table 1**. The coaches are specially trained to deliver the intervention, and they receive weekly supervision from two senior CBT therapists. Phone calls are recorded for quality control.

If the woman is eligible, she is asked to provide informed consent on a secure website. At the start of the baseline assessments, the women fill in the EPDS again to assess how stable their depressive symptoms are. If they score at least 9 points, they proceed to the full digital baseline assessment. If they score 8 points or less, they are excluded. A separate phone call is made after the baseline questionnaires. In this phone interview, the women are assessed using selected sections of the structured diagnostic interview, Mini-International Neuropsychiatric Interview (MINI) (29). The included sections cover depression, suicidality, bipolar disorder, and anxiety disorders. If the woman reports current suicidality, she is excluded from the study and referred to appropriate services. Other exclusion criteria include psychotic and bipolar disorders, substance abuse, active ongoing psychotherapy, participating in another intervention, and multiple pregnancy (**Table 1**). The women can be referred to local healthcare services if more individual treatment is considered necessary, and the coaches can also consult a psychiatry specialist at any time.

### 2.4 Intervention

*Stronger Together* is a targeted iCBT intervention for pregnant women with depressive symptoms who score ≥9 points on the EPDS. It is based on the *Coping with Depression* course, which has been effective in preventing and treating depression in several RCTs and has been implemented in different target populations and settings (30). The highly structured *Stronger Together* intervention includes basic CBT components: psychoeducation, behavioral activation, coping with social relationships, cognitive restructuring, and preventing setbacks (**Table 2**). It comprises seven-weekly themes on a digital platform and weekly phone coaching. There will also be two booster sessions, approximately 2 and 7 months after the birth, including a phone call and a digital component. No physical visits or face-to-face communication are included. The aim is to strengthen the expectant mother’s coping skills. The phone coaches are specially trained healthcare professionals who support and motivate the women. The weekly phone sessions are scheduled when they are most convenient for the women. To ensure the fidelity and quality of the intervention, the phone coaches receive weekly supervision by two experienced CBT therapists, including case consultations, intervention management, and support on the use of CBT principles during the coaching process.

**Table 2:** Core components of the stronger together iCBT intervention.

Theme	Key elements	Skills and tools
Introducing the intervention	Psychoeducation, CBT model, working alliance	Monitoring mood/mood diary and mood compass
1. Body sensations and mood	Healthy life habits, changes in body during pregnancy, body sensations	Relaxation, regular exercise/mood diary, mood compass
2. Behavior and mood	Behavioral activation	Listing pleasant activities/mood diary, mood compass
3. Improving mood through pleasant activities	Increasing behavioral activation	Planning ahead/mood diary, mood compass, activity schedule
4. Keeping up social relationships	Social networks	Asking for help/mood diary
5. Mood and thinking	Positive and negative thinking, negative cognitive bias	Recognizing positive and negative thoughts/mood diary, mood compass
6. Realistic thinking and self-compassion	Cognitive restructuring	Choosing positive thoughts/mood diary, mood compass
7. Preventing setbacks	Useful skills, monitoring mood diary, skills summary, future plans	Self-checking/mood diary, future plans

iCBT, internet-assisted cognitive behavioral therapy.

### 2.5 Educational control group

The control group receives access to digitally delivered psychoeducational material, which includes information about pregnancy-related life changes, social relationships, wellbeing, and healthy habits such as regular exercise, sleep, and coping with stress. The controls do not receive weekly phone coaching or access to the core CBT components. The controls receive standard prenatal care at the maternity clinics.

### 2.6 Adverse events

*Stronger Together* is a minimal-risk trial based on CBT, which is considered the first-line treatment for depression. CBT has no significant adverse effects, and we do not anticipate that the intervention will have negative side effects. Any adverse effects that do occur are handled during the weekly phone calls, and the women can also contact the study team at any time.

### 2.7 Primary outcome

The primary outcome variable is any change in the total EPDS score (31) from baseline at 13–18 weeks of gestation to 11 weeks after randomization. The EPDS is a standard 10-item self-report questionnaire, which was originally developed to screen for postpartum depression. It has been widely validated to detect depression both during (Cronbach’s α = 0.82–0.84) (32) and after pregnancy (Cronbach’s α = 0.87) (31). However, it has not been validated specifically in Finland. The respondents are asked about symptoms of depression in the past week. The score ranges from 0 to 30, and 13 points or above is normally used as a cutoff for depression during pregnancy. Because this study focuses on early intervention, we use a cutoff of 10 points on the total EPDS score to ensure that we include pregnant women with subclinical depressive symptoms (18, 33). The primary and secondary outcomes have been described in detail in **Table 3**.

**Table 3:** The schedule of enrollment, interventions, and assessments.

	Enrollment		Allocation	Post-allocation		
Time point		T0		Intervention period	T1	T2
Enrollment						
EPDS screen	X					
Informed consent	X					
Eligibility screen	X					
MINI interview	X					
Allocation			X			
Intervention						
iCBT intervention group				iCBT with telephone coaching		
Educational control group				Online psychoeducation		
Assessments						
*Depression*						
Symptoms of antenatal depression (primary outcome) EPDS		X				X
Depressive symptoms BDI-II		X				X
*Anxiety*						
Generalized anxiety GAD-7		X				X
Pregnancy-related anxiety PRAQ-R2		X				X
Social phobia SPIN		X				X
*Other measures*						
Maternal attachment MAAS		X				X
Perceived stress PSS		X				
Quality of life and sleep		X				X
*Treatment-related measures*						
Alliance WAI-SR					X	
Use of the digital content					X	
Satisfaction CSQ-I					X	
Biological samples						
Serum sample		X				
Buccal cell swab		X				X

T0 is baseline, T1 is immediately after the intervention, and T2 is 11 weeks after randomization.

EPDS, Edinburgh Postnatal Depression Scale; BDI-II, Beck Depression Inventory-II; GAD-7, Generalized Anxiety Disorder 7-Item Scale; MINI, Mini-International Neuropsychiatric Interview; PRAQ-R2, Pregnancy-Related Anxiety Questionnaire-Revised; SPIN, Social Phobia Inventory; MAAS, Maternal Antenatal Attachment Scale; PSS, Perceived Stress Scale; WAI-SR, The Working Alliance Inventory-Short Revised; CSQ-I, Client Satisfaction Questionnaire; iCBT, internet-assisted cognitive behavioral therapy.

### 2.8 Secondary outcomes

*Depression.* The Beck Depression Inventory-II (BDI-II) (34) is also used to examine depressive symptoms. It is a widely used 21-item self-report instrument measuring the severity of depression with high validity and reliability: Cronbach’s α is 0.88 when used during pregnancy and 0.89 among postpartum women (35). In Finland, the scale has been validated for adult populations (36). Each item is scored from 0 to 3, and the total score, which we will evaluate, ranges from 0 to 63. The BDI can be used to assess the nine symptoms of depression included in the Diagnostic and Statistical Manual of Mental Disorders, Fifth Edition (37).

*General anxiety.* The total score of the Generalized Anxiety Disorder 7-Item Scale (GAD-7) is used to assess changes in anxiety symptoms from baseline to follow-up. GAD-7 is a brief screening measure for generalized anxiety (38) with good reliability (Cronbach’s α = 0.89). It includes seven questions about anxiety symptoms in the last 2 weeks, and the frequency is rated from *not at all* to *nearly every day*. A total score of nine indicates potentially clinically significant anxiety symptoms. The GAD-7 has been validated for pregnant women (39) and for use in Finnish primary care settings (40).

*Pregnancy-related anxiety.* Changes in anxiety symptoms related to pregnancy are assessed using the second revision of the Pregnancy-Related Anxiety Questionnaire (PRAQ-R2) (41). This version can be administered to pregnant women regardless of parity. The scale has been implemented and validated in Finland and has shown sufficient reliability (Cronbach’s α = 0.71–0.85 depending on parity and pregnancy week) (41). It consists of 10 items with three subscales: fear of giving birth, worries about delivering a physically or mentally handicapped child, and concerns about the woman’s appearance. We will evaluate the total score and the three subscales separately.

*Social anxiety.* Changes in social anxiety symptoms are assessed using the total score of the Social Phobia Inventory (SPIN), which contains 17 items about avoidance and fear of embarrassment. Each item is rated on a scale of 0 to 4, with higher scores indicating greater distress. A result of 19 points or above on the SPIN is considered indicative of social phobia. The SPIN has demonstrated good test–retest reliability, internal consistency (Cronbach’s α = 0.82–0.94), and validity (42). In Finland, it has been validated for adolescents (43), but not for pregnant women.

*Stress.* The Perceived Stress Scale (PSS) (44) examines changes in personal stress levels. The 10-item scale covers stressful feelings and thoughts during the last month, ranging from 0 for *never* to 4 for *very often*. The total score, which we will evaluate, ranges from 0 to 40, with a higher score indicating greater perceived stress. Cronbach’s α for this instrument is between 0.84 and 0.86, and it has been validated for pregnant women (45). In Finland, it has been validated for measuring stress symptoms of undergraduate students (46).

### 2.9 Additional outcomes

*Maternal attachment*. The total score of a shorter 12-item version of the Maternal Antenatal Attachment Scale (MAAS) (47) is used to evaluate antenatal attachment. This scale focuses on maternal attitudes, thoughts, feelings, and behaviors toward the growing fetus, and higher scores indicate a more adaptive mother–infant bonding style. This scale has an internal consistency of 0.73 (48) and has not been validated in Finland.

*Sleep and quality of life.* Single questions regarding quality of life, loneliness, satisfaction with social relationships, and sleep are administered. Each question is analyzed separately.

### 2.10 Background measures

Sociodemographic and health-related data, including age, family structure, level of education, employment, and chronic diseases, are collected at baseline. This includes detailed information about previous psychiatric diagnoses and their treatment, smoking, substance abuse, and previous pregnancies and childbirth. Further, the women are asked about meaningful life events 12 months before the beginning of the study.

### 2.11 Biological samples

Biological samples are collected to explore possible biological mediators for therapeutic response. Maternal serum samples are collected from all mothers who give permission to use their previously collected samples at antenatal clinics for study purposes in the screening phase. Buccal cell swabs are collected from the women in mid-pregnancy. The polygenetic risk scores for depression will be calculated, and the association between the genetic risk scores and the treatment responsiveness will be explored. The inflammatory markers and epigenetic alterations will be determined by examining the differences in DNA methylomes based on the treatment responsiveness.

### 2.12 Treatment-related measures

Treatment-related measures are only administered to the intervention group.

*Therapeutic alliance.* The Working Alliance Inventory-Short Revised (WAI-SR) is used after the iCBT treatment. This instrument measures the therapeutic and collaborative relationship between the coach and the patient. It has shown good psychometric properties specifically for guided internet interventions (Cronbach’s α = 0.93) (49).

*The use of digital content.* The digital platform automatically collects user data on how long the woman spends on the website, which material she uses, and how often she reads it. The frequencies are daily/almost daily, a couple of times a week/once a week, or never. We also examine the proportion of completed coaching calls, the time interval between calls, and the call durations. The woman’s location online and during coaching calls is also recorded, namely, at home, at work, or in another place.

*Treatment satisfaction.* Women who receive the intervention are asked about the general usability and perceived benefit of the intervention after they finish the treatment. In addition, the structured Client Satisfaction Questionnaire for internet-based interventions (CSQ-I) is used. This tool has shown good psychometric qualities and reliability (McDonald’s Ω 0.93–0.95) for assessing general user satisfaction with Web-based interventions for depression (50).

### 2.13 Randomization and masking

Randomization is stratified by the study site, and it takes place after the baseline assessment and MINI interview are completed. The study statistician creates the randomization codes separately for each study site using the letters A and B for the intervention and control groups, respectively. The randomization sequences are generated with a 1:1 ratio using the SAS 9.4 computerized random permuted block sequence generator (SAS Institute, Cary, NC, USA) with concealed block sizes. The statistician stays blinded to the treatment groups until the randomization expert opens the coding after the blind review. After each subject is randomized, the platform unlocks the appropriate user interface, and the subjects receive an email telling them what group they are in. The statistician and randomization expert are part of the study team, but they are not directly involved in the conduct of the study.

### 2.14 Data management

All the data gathered by the digital platform are stored in a PostgreSQL database (The PostgreSQL Global Development Group). After the collection period has been completed, the data manager will ensure the accuracy of the data and then import the SAS datasets for statistical analysis and reporting. Metadata are stored in spreadsheets, and a data dictionary provides guiding principles for the dataset and variable construction, as well as study-specific information. All datasets from different data sources will be imported to SAS with programs that follow the guidelines in the data dictionary. Code books will be created to document the contents of the SAS datasets: variable names, labels, types, and formats. Quality control procedures such as database auditing, data reviewing, and reconciliation will be implemented to ensure that the statistical analyses use acceptable quality data.

All the baseline questionnaires are filled in online, and participants have to complete all the required fields. The platform does not allow them to select multiple responses to Likert-type questions. During the follow-up phase, participants are given the choice to complete the questionnaires online or on paper versions, which are manually digitized using Microsoft Access (Microsoft Corp., Redmond, WA, USA). To ensure consistency, the online templates follow a similar layout to the paper questionnaires. Personnel digitalizing these follow the guidelines on how to handle unclear answers, such as multiple answers selected. The collected data are stored on the University of Turku file servers, and access is limited to appointed researchers, statisticians, and the data manager. The files are automatically backed up each day and kept for 26 weeks.

### 2.15 Power

The aim of the power calculations was to determine how many subjects should be enrolled in the intervention and control groups to achieve 80% power to reject the null hypothesis of equal means. The power calculations were based on a population mean difference of μ1 − μ2 = 9.0 − 10.5 = −1.5 in EPDS scores, with a standard deviation of 6.30 for both groups and a significance level of α = 0.05. This resulted in 278 subjects in both groups. Because we anticipate 14% attrition in both groups, we aim to enroll 317 dyads in both groups. To promote participant retention, each subject will receive reminders by email and telephone from the study coaches. No further data will be collected if a participant chooses to withdraw from the study. If a participant discontinues the intervention but does not withdraw from the study, data will be collected according to the protocol.

### 2.16 Analysis plan

Statistical analyses will be carried out on the intention-to-treat dataset and repeated for the per-protocol dataset that consists of all evaluable subjects. Categorical variables will be presented as numbers and percentages, and continuous variables as means and standard deviations. Pearson’s chi-square tests or Fisher’s exact tests will be conducted to explore differences in categorical variables between the intervention and control groups at baseline. Two-tailed, two-sample t-tests will be used to explore differences in continuous variables between the groups at baseline. The primary outcome measure is the total EPDS score, which is collected at baseline and 11 weeks after randomization. The follow-up EPDS total scores and changes in EPDS scores will be analyzed using a linear mixed-effects model where the baseline EPDS total score and the stratifying factor study site are included as covariates. The follow-up EPDS total scores and changes in EPDS scores will be analyzed using analysis of covariance (ANCOVA), adjusted for the baseline EPDS total score and stratifying factor study site as a covariate. The secondary outcome measures will be analyzed using the same modelling approach as the primary outcome measure. If feasible, the study subjects will be included as a random effect to generalize the results beyond our study sample. The primary outcome will be analyzed blinded, as the treatment code will only be opened after the study statistician has analyzed the primary outcome. Two-sided significance levels of 0.05 and 95% confidence intervals will be used in statistical testing. The statistical analyses will be carried out using the SAS statistical software (SAS 9.4, SAS Institute, Cary, NC, USA).

## 3 Discussion

There is emerging evidence that iCBT interventions are feasible and acceptable treatment alternatives for antenatal depression. However, their efficacy is usually not well-documented. Most studies have been based on rather small sample sizes (19) and are not initiated alongside routine health check-ups.

The RCT described in this protocol uses a well-designed method and includes multiple sites across Finland. The structured *Stronger Together* intervention includes core CBT components on a digital platform: psychoeducation, behavioral activation, coping with social relationships, cognitive restructuring, and preventing setbacks. These themes are combined with weekly telephone coaching, which ensures human contact throughout the intervention. This hybrid approach ensures an equal treatment pathway for all intervention participants yet includes an individually tailored element. Finally, as a methodological strength, our RCT assesses the level of depressive symptoms using both self-report questionnaires and the MINI clinical interview. The potential limitations include the reliability of self-reporting and stigma-related factors, which may make it challenging to detect those pregnant women who need early intervention (51). Some studies have suggested that pregnant women who have depressive symptoms find it difficult to seek help and are concerned about whether they will be taken seriously (52, 53).

Antenatal depression presents potentially long-term detrimental consequences for both mother and offspring. *Stronger Togethe*r is an active iCBT intervention especially adapted for pregnancy. It aims to provide skills to cope with depressive symptoms and help to adapt to the new stage of life. It has the potential to have a positive impact on the offspring’s development by improving maternal mental health and mother–child interaction.

If the *Stronger Together* iCBT intervention proves to be effective, it has significant potential in the treatment of antenatal depression. It would be an evidence-based, low-threshold, and accessible treatment. Digital delivery offers a solution to overcome geographical, economic, and stigma-related barriers to seeking and receiving treatment.
